# Effects of Dl-3-n-butylphthalide on cognitive functions and blood–brain barrier in chronic cerebral hypoperfusion rats

**DOI:** 10.1007/s00210-023-02530-5

**Published:** 2023-05-27

**Authors:** Yang Ma, Shiling Chen, Yuanwei Li, Jiahui Wang, Jingfei Yang, Jie Jing, Xia Liu, Yunjie Li, Jingyi Wang, Ping Zhang, Zhouping Tang

**Affiliations:** 1 Department of Neurology, Tongji Hospital, Tongji Medical College, Huazhong University of Science and Technology, Wuhan, China; 2 Department of Neurology, Third Affiliated Hospital of Soochow University, Changzhou First People’s Hospital, Changzhou, China

**Keywords:** Vascular cognitive impairment, Chronic cerebral hypoperfusion, Blood–brain barrier, Pericyte

## Abstract

Vascular cognitive impairment (VCI) has been one of the major types of cognitive impairment. Blood–brain barrier damage plays an essential part in the pathogenesis of VCI. At present, the treatment of VCI is mainly focused on prevention, with no drug clinically approved for the treatment of VCI. This study aimed to investigate the effects of DL-3-n-butylphthalide (NBP) on VCI rats. A modified bilateral common carotid artery occlusion (mBCCAO) model was applied to mimic VCI. The feasibility of the mBCCAO model was verified by laser Doppler, ^13^N-Ammonia-Positron Emission Computed Tomography (PET), and Morris Water Maze. Subsequently, the Morris water maze experiment, Evans blue staining, and western blot of tight junction protein were performed to evaluate the effect of different doses of NBP (40 mg/kg, 80 mg/kg) on the improvement of cognitive impairment and BBB disruption induced by mBCCAO. Immunofluorescence was employed to examine the changes in pericyte coverage in the mBCCAO model and the effect of NBP on pericyte coverage was preliminarily explored. mBCCAO surgery led to obvious cognitive impairment and the decrease of whole cerebral blood flow, among which the blood flow in the cortex, hippocampus and thalamus brain regions decreased more significantly. High-dose NBP (80 mg/kg) improved long-term cognitive function in mBCCAO rats, alleviated Evans blue leakage and reduced the loss of tight junction proteins (ZO-1, Claudin-5) in the early course of the disease, thereby exerting a protective effect on the blood–brain barrier. No significant changes in pericyte coverage were observed after mBCCAO. High-dose NBP improved cognitive function in mBCCAO rats. High-dose NBP protected the integrity of BBB by upregulating TJ protein expression, rather than regulating pericyte coverage ratio. NBP could be a potential drug for the treatment of VCI.

## Introduction

Dementia is a clinical syndrome characterized by cognitive impairment and diminished ability to perform daily activities, with clinical symptoms including decreased learning, memory and language abilities, diminished intelligence, behavioral abnormalities, and personality changes (Arvanitakis et al. 2019). Vascular dementia (VaD), the second most common type of dementia only after Alzheimer's disease (AD), is an irreversible condition mainly attributable to various systemic vascular or cerebrovascular diseases (Sun 2018). In recent years, the term “vascular cognitive impairment (VCI)” becomes more commonly used, which is a general term that can be classified according to the illness severity, from mild cognitive impairment to the most severe aspects of this disease, VaD (Skrobot et al. 2018; van der Flier et al. 2018).

Chronic Cerebral hypoperfusion (CCH), a decreased cerebral blood flow (CBF) state caused by various cerebrovascular diseases, is now regarded as a major contributor to VCI and VaD (Duncombe et al. 2017). Although the pathological mechanism of VCI is still not fully understood, there is considerable evidence indicating that endothelial dysfunction assumes a fundamental part in the pathogenesis of VCI (Wang et al. 2018). CCH may cause endothelial dysfunction, and then endothelial dysfunction leads to decreased autoregulation ability and blood–brain barrier (BBB) integrity of small arteries and capillaries, which further exacerbates local short- or long-term cerebral hypoperfusion (Washida et al. 2019). Thus, endothelial dysfunction may be a target to stop this vicious cycle.

DL-3-n-butylphthalide (NBP) is a synthesized compound developed from the extract of celery seeds, containing L- and D-isomers of butylphthalide. NBP has been widely used as a neuroprotective treatment for ischemic stroke in China, and the Phase II clinical trial (NBP in Adult Patients With Acute Ischemic Stroke, NCT02905565) permitted by the United States Food and Drug Administration has been completed. It has been shown that NBP exerts neuroprotective effects in ischemic stroke by promoting angiogenesis, improving local blood flow in the ischemic region, decreasing the level of reactive oxygen species and suppressing inflammatory responses (Yan et al. 2017; Qin et al. 2019; Zhou et al. 2019). Multicenter random control trial showed that NBP treatment for 6 months improved cognitive function in patients with subcortical VCI without dementia (Jia et al. 2016). However, the specific mechanism of NBP improving cognitive impairment needs to be further studied.

It had been proven that NBP may exert a direct protective effect on endothelial cells in various disease models. NBP suppresses oxidative/nitrosative stress and mitochondrial damage in endothelial cells in the oxygen–glucose deprivation model and MPP^+^-induced cellular model of Parkinson’s disease (Huang et al. 2010; Liu et al. 2017). NBP has also been reported to eliminate brain edema and BBB damage in rats after focal cerebral infarction induced by the photochemical method (Hu et al. 2014). Thus, it is hypothesized that NBP may alleviate endothelial dysfunction induced by CCH.

In this study, we used a modified two-step bilateral common carotid artery occlusion (mBCCAO) model to mimic the CCH state. Laser Doppler, ^13^N-Ammonia-PET, and Morris water maze were conducted to evaluate changes in cognitive function and CBF in the mBCCAO model. Then, we detected the protective effects of different doses of NBP on cognitive function and BBB integrality in mBCCAO rats, and whether NBP attenuates blood–brain barrier damage by affecting the pericytes coverage ratio around endothelial cells.

## Methods

### Animals

Healthy adult male Sprague–Dawley (SD) rats, aged 6–8 weeks and weighing 250-300 g, were purchased from The Disease Control and Prevention Center of Hubei Province. The SD rats were housed for 1 week to acclimate to the environment before the formal experiment. Rats were kept in an SPF room (20–26 °C temperature; 40–65% humidity) on a 12 h light/12 h dark cycle, with enough food and water.

### Study design and grouping

**Fig. 1:**
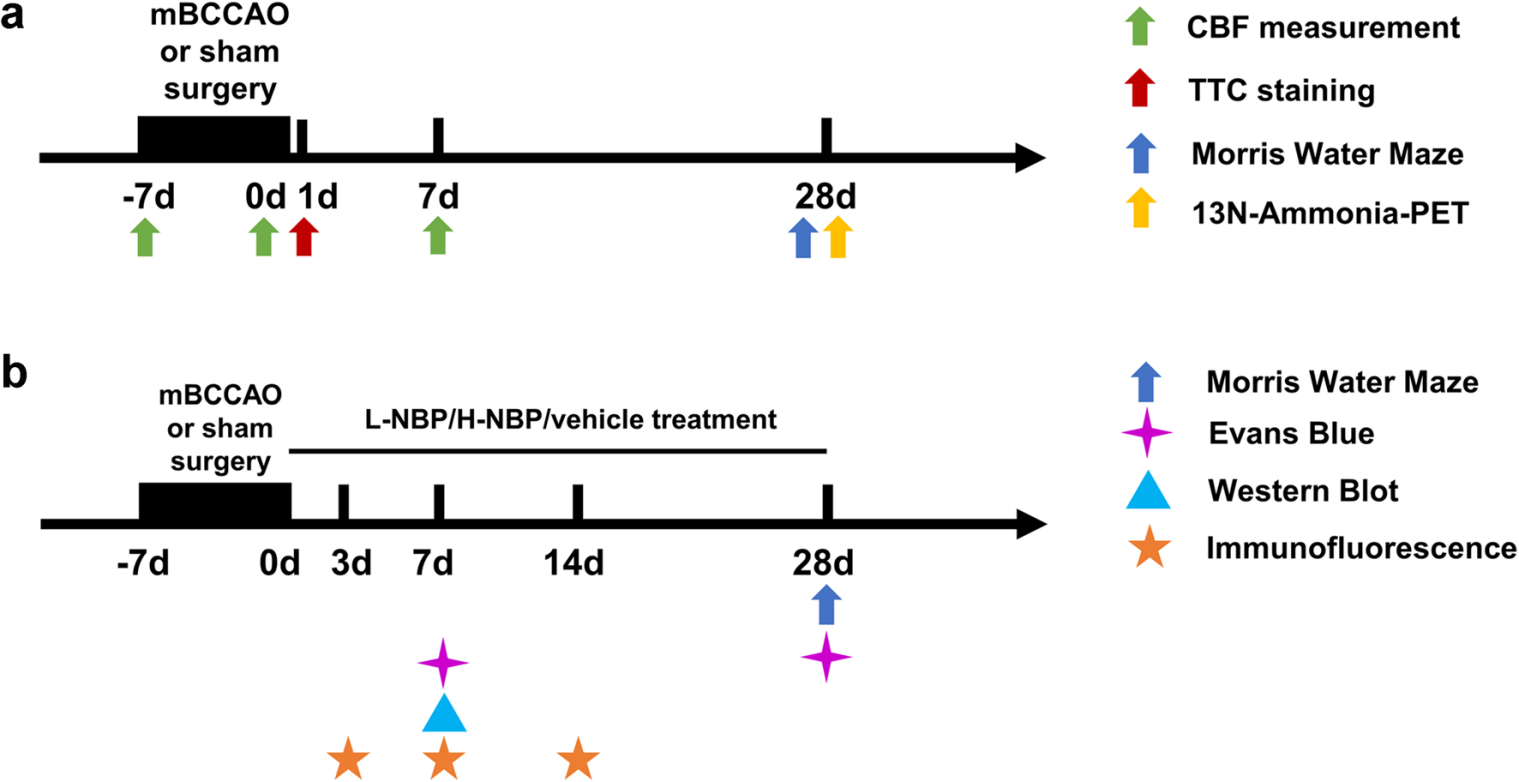
Study design. (**a**) For verifying the effect of mBCCAO modeling, SD rats were randomly divided into two groups: sham group and model group. TTC staining was conducted on the day after mBCCAO. CBF change was monitored by laser Doppler flowmeter during the whole mBCCAO or sham surgery process and 1 week after surgery. Morris Water Maze experiment and ^13^N-Ammonia-PET scan were conducted 28 days after mBCCAO; (**b**) For evaluating the therapeutic effect of NBP on mBCCAO rats, SD rats were randomly divided into 4 groups: sham group, model group, low-dose butylphthalide group (L-NBP group) and high-dose butylphthalide group (H-NBP group). Cognitive function was evaluated by the Morris Water Maze experiment 28 days after surgery, Evans blue leakage experiment was conducted on 7 days and 28 days after mBCCAO to examine the BBB damage. Quantitative analysis of tight junction protein by Western blot on 7 days after surgery, as well as pericyte coverage by immunofluorescence on 3, 7 and 14 days, were conducted to explore the mechanism of NBP’s protective effect on mBCCAO rats

For verifying the effect of mBCCAO modeling, SD rats were randomly divided into two groups: sham group and model group. The sham group only received the surgery that separated the carotid artery and vagus nerve. The model group received the mBCCAO surgery. CBF measurement, TTC staining, Morris Water Maze, and ^13^N-Ammonia-PET were conducted at different time points to evaluate the CBF change and cognitive function change after mBCCAO (Fig. 1a).

For evaluating the therapeutic effect of NBP on mBCCAO rats, SD rats were randomly divided into 4 groups: sham group, model group, low-dose butylphthalide group (L-NBP group), and high-dose butylphthalide group (H-NBP group). After mBCCAO surgery, the L-NBP group and H-NBP group rats were fed a dose of NBP (dissolved in corn oil) at the concentration of 40 mg/kg or 80 mg/kg by gavage. The sham group and model group were fed an equal volume of corn oil. Morris Water Maze, Evans blue leakage experiment, Western blot for tight junction protein, and immunofluorescence for pericyte coverage were conducted at different time points to evaluate NBP’s effect on cognitive function and BBB integrity after mBCCAO (Fig. 1b).

### mBCCAO surgery

mBCCAO surgery was conducted by an experienced researcher. The rats were anesthetized with isoflurane and then fixed in the supine position on a heating pad. After disinfection, the skin was incised through the median cervical line, and the subcutaneous tissue was slowly separated. Then, the right common carotid artery (RCCA) and vagus nerve were carefully isolated after exposing the right carotid sheath. In the model group, the RCCA was fully separated and then occluded by double ligation with two 4–0 sutures, while in the sham group the operation procedure was the same, but only the vessels were separated without ligation. During the operation, the respiratory and heart rate of the rats were observed, the incision was sutured after no obvious bleeding, and erythromycin ointment was applied to prevent infection. The rats were kept warm until they were awake from anesthesia and then sent to separate ventilated cages for raising. One week after right common carotid artery occlusion (RCCAO), left common carotid artery occlusion (LCCAO) was performed in the same way, and the rats in both groups were kept in the same condition after the operation.

### TTC staining

After the animals were sacrificed, the brains were quickly taken out, placed in phosphate buffer saline (PBS) at 0–4 °C for transfer, and frozen at -20 °C for 30 min. Then we cut brain slices with a thickness of 2 mm, put the slices in a 2% red tetrazolium solution (Servicebio, Wuhan, China) in a water bath at 37 °C for 30 min in the dark, and shake the container slightly every 5 min to fully stain. The brain slices were taken out, washed with PBS solution for 3–5 min, and photographed immediately.

### CBF measurement

The moorVMS-LDF contact laser Doppler flowmeter (Moor, UK, with two channels) was used for CBF measurement. The rats’ skull was exposed and the area to be examined was slightly thinned, and the laser Doppler flow (LDF) measurement microtip optic fiber probes were fixed to the point on the skull (1 mm posterior and 5 mm left anterior to bregma respectively) with quick-drying glue. The rats were then rotated to a supine position and CBF was continuously recorded by moorVMS-LDF contact laser Doppler flowmeter during the RCCAO and LCCAO operation and 1 week later.

### Morris water maze

The Morris water maze experiments were performed and analyzed by two persons blinded to the treatment of the animals. Two days before starting the experiment, the rats were transferred to a rack outside the water maze room and given a normal diet to remove any existing directional olfactory or auditory information. The rats were transferred to the water maze room 2 h before the start of the training, allowing them to adapt to the indoor environment. The water was stained black by ink, with the temperature adjusted to 22 ± 1 °C. The light intensity was 40 lx. The swimming trajectory of the rats in the water maze was recorded with the Morris Imaging System. The platform was placed 1.5 cm below the surface of the water. On the 1–4 day, from the other three quadrants except for the quadrant in which the platform is located, the rats were gently placed into the water with their heads facing the pool wall. Escape latency and the total distance of swimming were recorded. If the rats could find the platform within 60 s, they would be permitted to stay on the platform for 10 s. If they could not find the platform, their escape latency would be recorded as 60 s, the rats were led to the platform and remained there for 15 s. Every day, each rat was trained four times, taking the average of 4 training sessions as the spatial learning ability score of the day. On day 5, the platform in the water maze was removed. The rats were released from the opposite quadrant of the original platform, being allowed to swim freely to explore the pool for 60 s. Then we recorded the time that rats spent in the original quadrant where the platform was, and the number of times crossing the original platform location.

### ^13^N-Ammonia-Positron Emission Computed Tomography (PET)

The small-animal PET/CT scanner (Supernova, China) was used to quantify cerebral blood flow 4 weeks after surgery. The rats were placed into a rat holder compatible with the PET acquisition systems, and anesthetized with isoflurane during the whole process. 24 gauge catheters were used for the administration of ^13^N-Ammonia via tail veins. ^13^N-Ammonia was injected synchronously at the beginning of sequence acquisition**.** PET scan images were acquired in the 400–700 keV energetic window. CT acquisitions were performed after each PET scan, providing anatomical information about each animal to facilitate the later image reconstruction. PET images were analyzed using PMOD image analysis software (PMOD Technologies Ltd., Switzerland). For the whole brain, regions of interest (ROIs) were defined along the brain border. For specific brain regions such as frontal, motor, somatosensory and insular cortices, hippocampus, thalamus and cerebellum, ROIs were automatically generated by the MRI rat brain atlas provided by the software. For quantitative evaluation, the standardized uptake values (SUV) were calculated by dividing the tissue activity by the injected dose of radioactivity per unit body weight.

### Measurement of BBB permeability

On days 7 and 28 after surgery, the rats were injected with 2% Evans blue (Biofroxx, Germany) via tail vein at a dose of 40 mg/kg. 2 h later, the intravascular Evans blue (EB) was removed by cardiac perfusion with PBS. The brains of the rats were taken out by cervical dissection after anesthesia by pentobarbital sodium. Then the cerebellum and low brainstem were removed, preserving only two cerebral hemispheres. The brain tissue was then stored in formamide (protected from light) at room temperature for 2 days. The absorbance of the supernatant was measured after centrifugation at 3000 rpm at 4℃, and the EB level of the brain tissue was calculated by spectrophotometry (wavelength 620 nm) based on the Evans blue standard curve, reflecting the leakage of BBB.

### Western blot analysis

The rats were euthanized with pentobarbital sodium (100 mg/kg, i.p.). Meninges were dissected and brain tissues in the cortex (about 50 mg) were homogenized in radioimmunoprecipitation assay (RIPA) buffer (Beyotime, China) containing 1% phenylmethanesulfonyl fluoride (PMSF) using a grinding instrument(Servicebio, China). Protein concentration was detected using a bicinchoninic acid protein assay kit (Beyotime, China). 20 µg of protein from each sample were loaded on 8% or 10% SDS-PAGE gels and separated by electrophoresis. The separated proteins were transferred to polyvinylidene difluoride membranes. After being blocked with 5% skimmed dry milk for 1 h at room temperature, the membranes were incubated with the rabbit anti-Claudin5 antibody (49,564, Cell Signaling Technology, USA), rabbit anti-ZO-1 antibody (13,663, Cell Signaling Technology, USA) on a shaker overnight. After 3 times washing with TBST for 5 min each and then incubation with the secondary antibodies (HRP Goat Anti-Rabbit IgG (H + L), AS014, Abclonal, China) diluted with TBST on a shaker at 37 °C for 1 h, the membranes were washed with TBST 3 times for 5 min each again. Protein bands were visualized by enhanced chemiluminescence (ECL). The intensity of bands was detected by Image J software. The relative expression of each protein was quantified with the expression of β-actin, and each group was normalized with the Sham group.

### Pericyte coverage

#### Immunofluorescence

The pericyte coverage ratios on days 3, 7, and 14 after modeling were measured. After anesthesia with isoflurane, and perfusion with PBS solution at 4 °C and 4% paraformaldehyde through the heart, the whole brain was removed for Frozen sections. 20um Frozen brain sections were prepared by the freezing microtome(CM-1900, Leica, Germany). The sections of each group were incubated overnight with anti-PDGFRβ antibody(rabbit IgG, abcam, ab32570, 1:100, pericyte marker) and anti-CD31 antibody(goat IgG, R&D System, AF3628, 1:50, endothelial marker) and rinsed in PBS three times (5 min/time), and secondary antibodies (Alexa Fluor 488-conjugated AffiniPure Donkey anti Goat antibody, CY3-conjugated Donkey anti Rabbit antibody) were added to the sections. After 1 h incubation, secondary antibodies were washed. Finally, tissue sections were added with Antifade Mounting Medium with DAPI (Beyotime) and covered with glass.

#### Immunofluorescence analysis

All images were obtained with the OLYMPUS Fluorescence Microscopy (BX53). Images were taken from the right motor cortex. The pericytes coverage ratio was determined by the ratio of PDGFRβ positive pericytes around the capillaries to CD31 positive capillaries (≤ 10 um in diameter). Two blinded, independent observers counted the pericytes coverage ratio with the help of Adobe Photoshop software.

### Statistical analysis

The data in this article were analyzed statistically using SPSS19.0 (SPSS Inc., Chicago, III., USA), and expressed as mean ± standard error ($\overline{x }$±SEM). Controlled experiments with two groups were processed with a two-tailed unpaired t-test. As for one-way variable experiments including more than two groups, we used one-way ANOVA statistics and Tukey test for multiple comparisons between groups. The water maze experiment was analyzed using repeated-measures ANOVA, and the results of the spatial exploration experiment were statistically analyzed by two-way ANOVA and Bonferroni posttests. *P* < 0.05 was considered statistically different, and all experimental results were plotted using Graphpad Prism 9.0 software.

## Results

### CBF change after mBCCAO

**Fig. 2:**
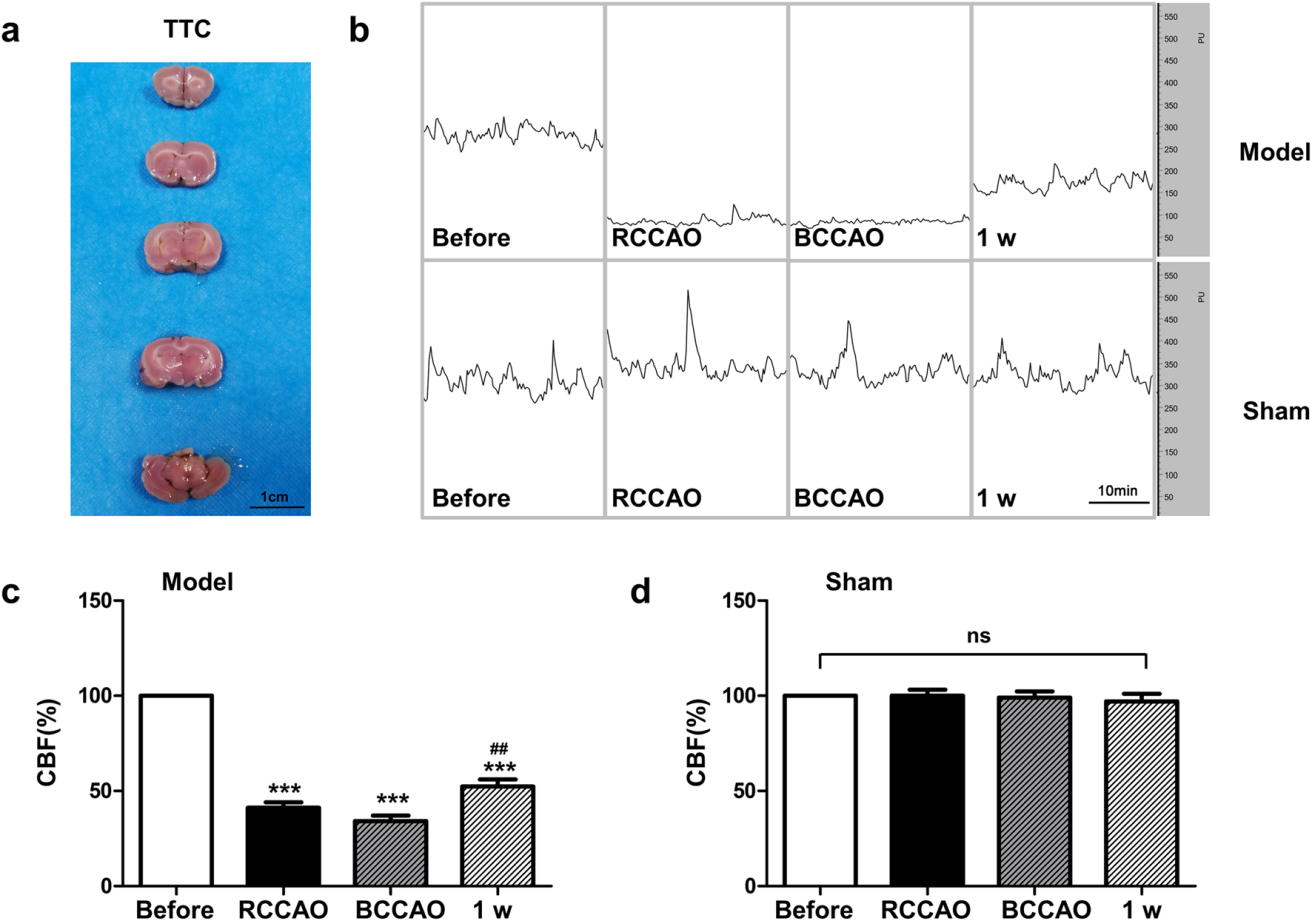
CBF change after mBCCAO. (**a**) TTC staining, 24 h after modeling by mBCCAO; (**b**) schematic diagram of cerebral blood flow measured by laser Doppler (time bar = 10 min); (**c**) changes of CBF in the model group during operation and 1 w after BCCAO; (**d**) changes of CBF in the sham group during the same period. ***RCCAO and BCCAO group compared with the condition before the operation, *P* < 0.001. ##BCCAO group compared with the condition 1w after operation, *P* < 0.01. ns: indicates no statistical difference, n = 6 for each group. Values are expressed as ($\overline{x }$±SEM)

Laser Doppler was applied to monitor the change of cortical CBF in rats during the operation. In the schematic diagram measured by laser Doppler, the CBF in the model group significantly decreased after the ligation of the right and both common carotid arteries (Fig. 2b and c, before vs RCCAO, *P* < 0.001; before vs BCCAO, *P* < 0.001), which is about 40% of the pre-operation baseline. TTC staining 24 h after the mBCCAO operation showed no infarct foci, which indicated that the mBCCAO could be a successful CCH rat model without the formation of cerebral infarct foci (Fig. 2a). One week after the surgery, CBF was elevated and statistically different from the data just after modeling (Fig. 2c, BCCAO vs 1w, *P* < 0.01) although still far from the pre-modeling levels, whereas the CBF of rats in the sham group was not significantly different at any time point (Fig. 2d).

### The cognitive impairment after mBCCAO

**Fig. 3:**
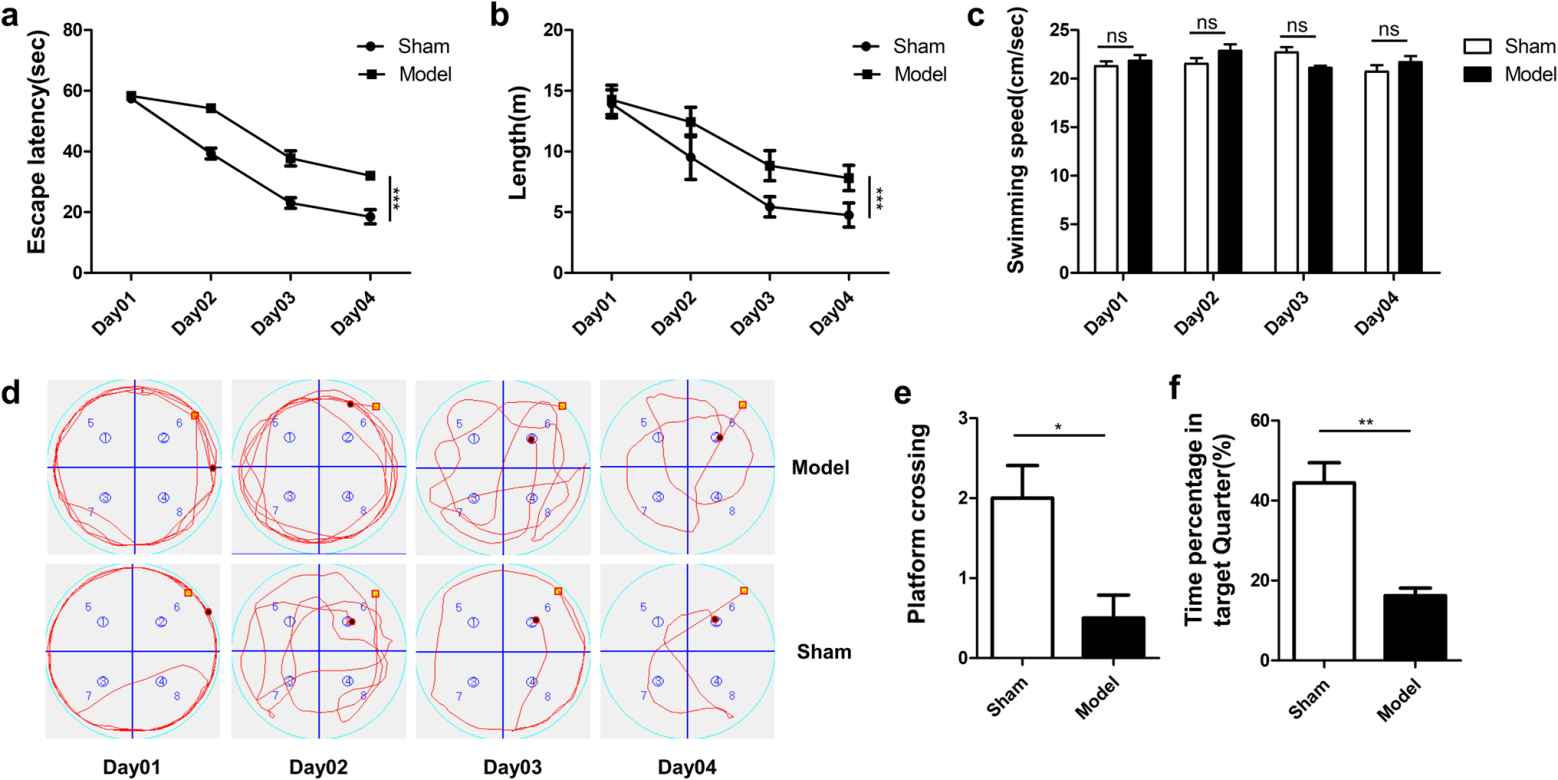
The cognitive impairment after mBCCAO. (**a**) The spatial learning ability of the rats in each group was compared by evaluating their average escape latency to reach the target platform on every training day; (**b**) the total distance each group swam during the training period, which is also an indicator of spatial learning ability; (**c**) the average swimming speed of each group; (**d**) the representative movement trajectories of the two groups searching for the target platform; (**e**) the number of times rats in each group crossed the target platform (removed) on the last day of the spatial exploration experiment to test their spatial memory ability; (**f**) the percentage of time spent searching for the removed platform in the target quadrant for the two groups. n = 6, *P < 0.05, **P < 0.01, ***P < 0.001, ns indicates no statistical difference. Values are expressed as ($\overline{x }$±SEM)

Four weeks after mBCCAO and sham operation, the Morris Water Maze test was performed to evaluate the learning and memory ability of the rats in two groups. During the first 4 days of training, the escape latency of the model group on day 4 was significantly higher than that of the sham group (Fig. 3a, P < 0.001), also with longer total traveling distance during the training period (Fig. 3b, P < 0.001), suggesting that the sham group had a better spatial learning ability. And there was no significant difference in the daily swimming speed of these two groups (Fig. 3c). The exploration trajectories of the rats in each group also showed a difference. The exploration paths of the sham group gradually moved from the "edge type" to the "middle type", while the model group rats moved less regularly (Fig. 3d). However, by counting the time that the rats were close to the walls (data not shown), we found no difference between two groups, which indicated that anxiety didn’t play a part in the exploration pattern. In the spatial exploration experiment on day 5, the number of times rats crossed the target platform (which has already been removed) and the proportion of time spent exploring in the target quadrant was recorded, and the difference between the two groups was statistically significant (Fig. 3e, P < 0.05, Fig. 3f, P < 0.01), indicating that the spatial memory ability of the rats in model group was impaired. Hence, it can be concluded that the CCH state after the mBCCAO leads to cognitive deficits.

### Quantitative measurement of CBF change by ^13^N-Ammonia-PET in different in various brain regions of mBCCAO rats

**Fig. 4:**
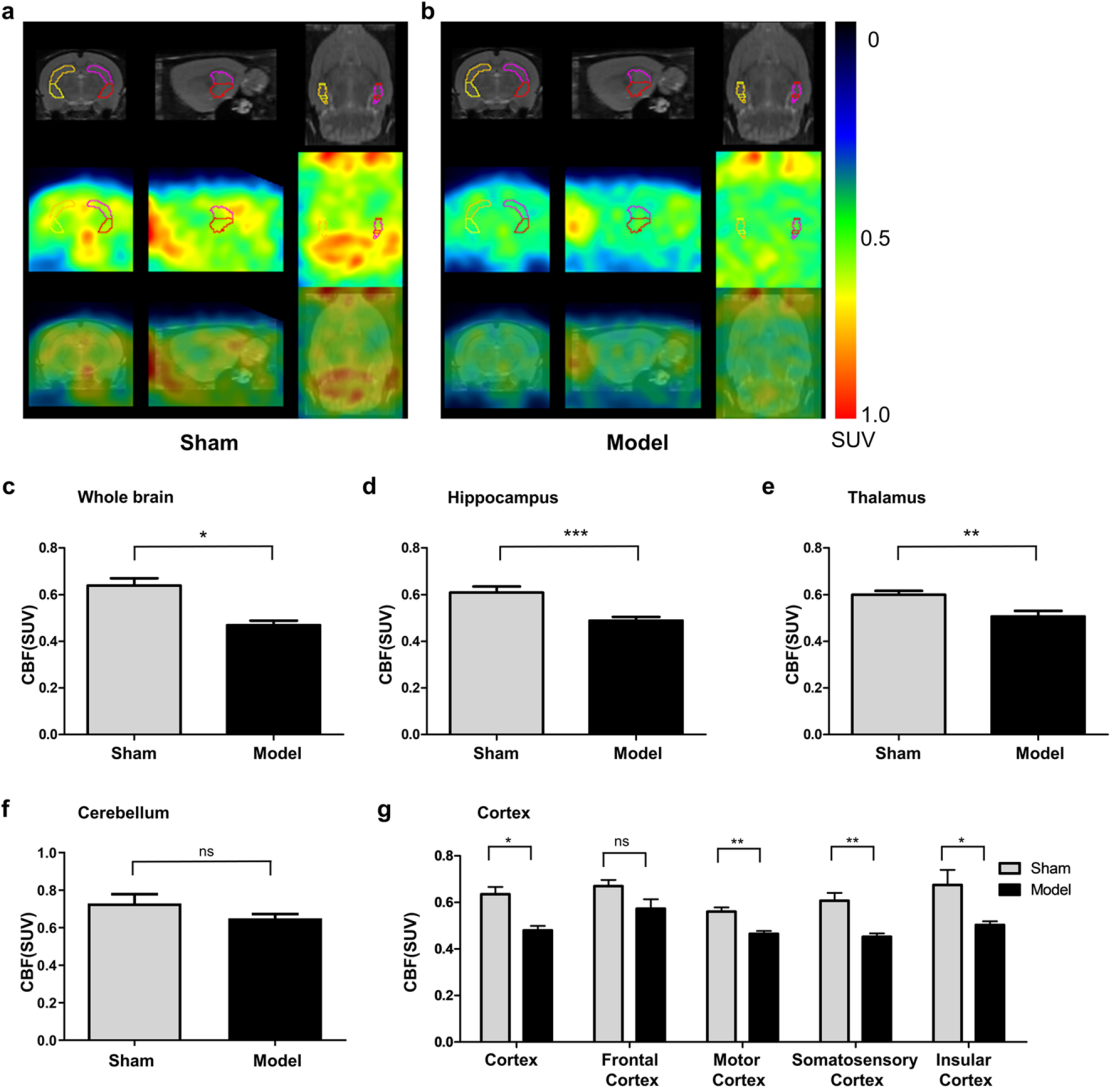
Quantitative analysis of CBF in the whole brain and different brain regions in the sham and model group. (**a, b**) The pseudo-color maps were derived from ^13^N-Ammonia-PET imaging and showed representative whole-brain perfusion in the sham (**a**) and model group (**b**) respectively. The red signal in the pseudo-color map represents the hyperperfusion state, while blue or green represents the hypoperfusion state. The circles represent the interested areas selected for CBF measurement; (c-g) The bar graphs represent the quantitative results of CBF in the whole brain (**c**), hippocampus (**d**), thalamus (**e**), cerebellum (**f**), and different parts of the cortex (**g**) in the two rat groups. The model group (n = 3) compared with the sham group (n = 3), *P < 0.05, **P < 0.01, ***P < 0.001, ns indicates no statistical difference. Values are expressed as ($\overline{x }$±SEM)

The ^13^N-Ammonia-PET technique was employed to measure the CBF change in different brain regions after modeling. The color signals of the images (strongest in red and weakest in blue) reflected the CBF changes in the whole brain (Fig. 4a). In the sham group, red and yellow signals appeared in many observed regions, whereas the pseudo-color images of the brain perfusion in the mBCCAO rats were largely dominated by green and blue. Quantitative analysis also showed that 4 weeks after modeling, mBCCAO rats had a decrease in CBF compared to the sham group (Fig. 4c, 0.47 ± 0.02 vs 0.64 ± 0.03, *P* < 0.05). Notably, the changes of blood flow in different brain regions are various. In the hippocampal region, CBF was significantly lower in the model group than in the sham one (Fig. 4d, P < 0.001). This trend was seen in the thalamus as well (Fig. 4e, P < 0.01), however, there was no significant difference in cerebellar blood flow in these two groups (Fig. 4f, P = 0.217). The standard uptake value (SUV) of blood flow in the cerebral cortex in the model group was 0.48 ± 0.02, which was lower than the sham group value(0.63 ± 0.03, *P* < 0.05). After separating the different parts of the cortex apart for quantitative analysis, we could find that the CBF decreased significantly in the motor and sensory cortex (*P* < 0.01), while there were no statistical differences in other cortices such as the frontal cortex (*P* = 0.121). It can be concluded that motor and sensory cortices were more susceptible to CCH than other parts of the cortex in mBCCAO rats (Fig. 4g).

### High-dose NBP improved cognitive function in CCH rats

**Fig. 5:**
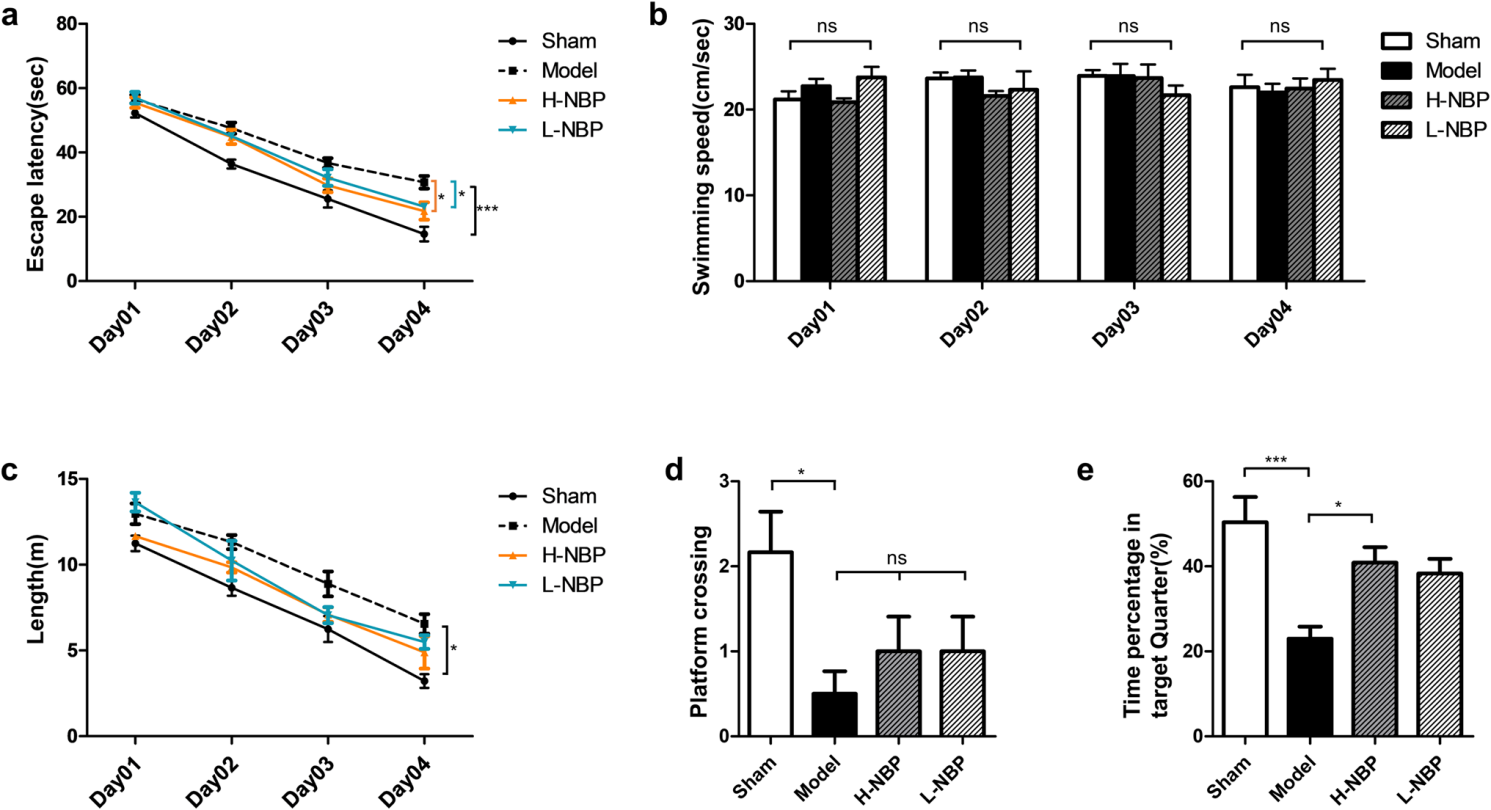
High-dose NBP improved cognitive function in CCH rats. (**a**) The spatial learning ability of rats in each group was reflected by comparing their average escape latency to the target platform; (**b**) the average swimming speed of each group on every training day; (**c**) the total swimming distance of each group during the training period; (**d**) the number of times rats crossing the target platform (removed) on the last day of spatial exploration experiment; (**e**) the percentage of time spent searching for the removed platform in the target quadrant for each group. n = 4–8, *P < 0.05, **P < 0.01, ***P < 0.001. ns indicates no statistical difference. Values are expressed as ($\overline{x }$±SEM)

Four weeks after the intervention with different doses of NBP, the Morris water maze experiment was performed to evaluate the learning and memory ability of rats. During the first 4 days of training, the escape latency of the model group was still significantly higher than that of the sham group (Fig. 5a, sham vs model, *P* < 0.001), and had a longer total traveling distance (Fig. 5c, sham vs model, *P* < 0.05), which was consistent with the previous results (Fig. 3a, b). After four weeks of continuous NBP gastric gavage, statistical differences in escape latency were found among the high-dose NBP group, the low-dose NBP group and the model group, with the difference being more significant between the high-dose NBP group and the model group (Fig. 5a, H-NBP vs model, *P* < 0.01; L-NBP vs model, *P* < 0.05). Statistical analysis of the daily swimming speed of the rats in each group did not reveal any significant difference (Fig. 5b), excluding the possibility that the difference in results was due to different swimming speeds. To sum up, the spatial learning ability of rats was significantly decreased after mBCCAO, and the application of NBP could improve the spatial learning ability of CCH rats to a certain extent. In the spatial exploration experiment on day 5, the sham group outperformed the model group with statistically significant results (Fig. 5d, sham vs model, *P* < 0.05; Fig. 5e, sham vs model, *P* < 0.001). The high-dose NBP group spent more time exploring the corresponding target quadrant than the model group (Fig. 5e, H-NBP vs model, *P* < 0.05), while no significant difference was found between the low-dose NBP group and the model group. With all the results, it had been proved that high-dose NBP intervention can significantly ameliorate the cognitive dysfunction of CCH rats and improve their spatial learning and memory abilities, while the effect of low-dose NBP intervention seems to be more limited.

### High-dose NBP ameliorated early BBB disruption in CCH rats

**Fig. 6:**
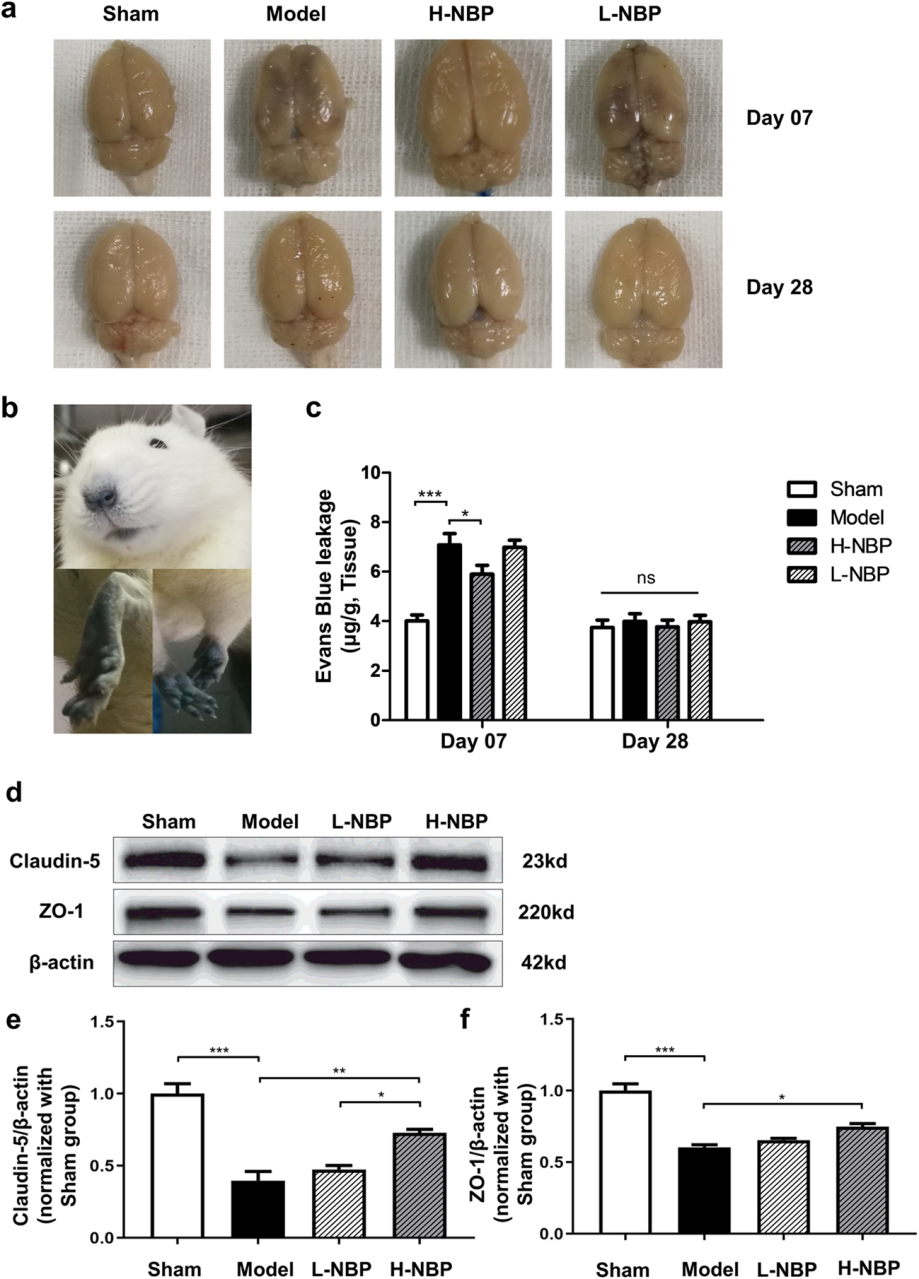
High-dose NBP ameliorated early BBB disruption in CCH rats. (**a**) EB leakage of rat brains in each group on day 7 and day 28 after surgery; (**b**) EB dye injection 2 h later, the eyes, ears, and upper and lower limbs of experimental rats turned blue; (**c**) EB leakage levels in brain tissue of rats in each group on day 7 and day 28 after surgery; (**d**) western blot representative bands of Claudin-5 and ZO-1; (**e**) quantitative analysis of Claudin-5 protein in each group; (**f**) quantitative analysis of ZO-1 protein in each group. For each group n ≥ 3, *P < 0.05, **P < 0.01, ***P < 0.001, ns indicates no statistical difference. Values are expressed as ($\overline{x }$±SEM)

On 7 and 28 days after the surgery, Evans blue leakage experiment was conducted to measure BBB permeability. The eyes, ears, and extremities of the rats gradually turned blue after injection of 2% EB through the tail vein (Fig. 6b). Two hours later, the brains were taken out after the heart perfusion. There was still relatively obvious EB leakage 7 days after mBCCAO, but after 28 days, EB leakage was no longer significant (Fig. 6a). Quantitative analysis showed no detectable EB leakage in the sham group, which had a statistical difference with the model group on day 7 (Fig. 6c, sham vs model, *P* < 0.001). The difference between the high-dose NBP group and the model group on day 7 was statistically significant (Fig. 6c, H-NBP vs model, *P* < 0.05), while low-dose NBP failed to improve the BBB condition, In contrast, the difference in EB leakage in each group had no statistical significance on day 28, which was just the same as that in the sham group (Fig. 6c), indicating the CCH rats no longer had significant EB extravasation as time progressed.

The expression levels of tight junction (TJ) proteins Claudin-5 and ZO-1, which are important for the integrity of BBB, were measured to further assess the effect of NBP on BBB. Western blot analysis showed that the expression of ZO-1 and Claudin-5 was significantly reduced in the model group compared with the sham group at 7 days after mBCCAO (Fig. 6d, f, model vs sham, *P* < 0.001). In the high-dose NBP group, the expression level of Claudin-5 was increased compared with the model group (Fig. 6e, H-NBP vs model, *P* < 0.01). The same trend was observed in the expression level of ZO-1 protein (Fig. 6f, H-NBP vs model, *P* < 0.05). Although the expression level of Claudin-5 and ZO-1 also increased slightly in the low-dose NBP but was statistically not significant (Fig. 6e, f, L-NBP vs model, *P* > 0.05).

### Pericyte coverage didn’t change after mBCCAO

**Fig. 7:**
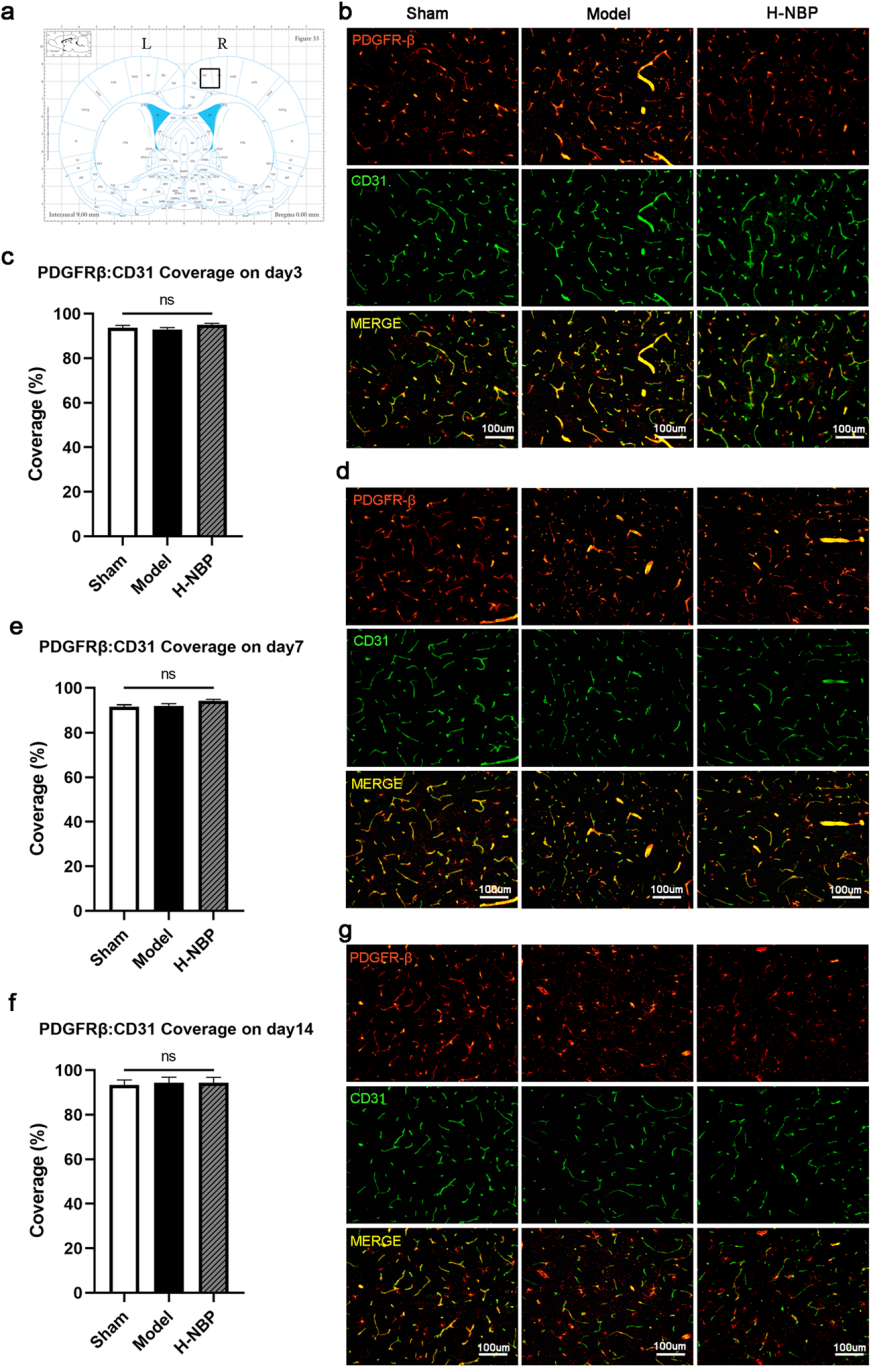
The changes of pericyte coverage in the cortex on days 3, 7, and 14 post-modeling. (**a**) The observation field was selected as the right motor cortex on the level near bregma; (**b**) Pericyte and vascular staining on sham, model, H-NBP rats on day3; (**c**) Quantitative analysis of the average ratio of PDGFRβ:CD31 on day3; (**d**) Pericyte and vascular staining on control, model, H-NBP rats on day7; (**e**) Quantitative analysis of the average ratio of PDGFRβ:CD31 on day7; (**f**) Quantitative analysis of the average ratio of PDGFRβ:CD31 on day14; (**g**) Pericyte and vascular staining on control, model, H-NBP rats on day14. For each group n = 3–6, ns indicates no statistical difference. Values are expressed as (x ® ± SEM). Scale bar: 100um

Pericytes are an important part of the neurovascular unit and play an important role in maintaining microcirculation blood flow stability and blood–brain barrier integrity. Decreased pericyte coverage ratio in the corpus callosum or cortex has been observed in various animal models of CCH, e.g. bilateral common carotid artery stenosis (BCAS) and BCCAO in previous studies. This may herald chronic ischemia leading to a decrease in pericytes number and impairment of their regulation roles in the blood–brain barrier and microcirculation. We then examine the cortex pericyte coverage ratio of each group at different time points. However, the result indicated that on days 3, 7, and 14, the pericyte coverage ratio showed no difference between each group (Fig. 7).

## Discussion

In this study, the effect and possible mechanism of NBP on the improvement of cognitive function in mBCCAO rats were explored. The experimental results proved that NBP improved the performance of CCH rats in the Morris water maze, and its amelioration of cognitive function may be achieved by protecting the integrity of BBB.

In our study only male rats were used, as studies have shown that estrogen and its derivatives might have a protective effect on CCH-induced neural injury. Xu et al. reported that 17β-estradiol alleviates endoplasmic reticulum stress injury in the hippocampus induced by CCH (Xu et al. 2021). 17β-estradiol is capable to prevent BCCAO-induced hippocampal spine loss and improve cognitive function (Zhu et al. 2017b).

Rats own similar cerebral vascular structures to humans, such as the circle of Willis, making them the idealized animals to mimic chronic ischemic states. BCCAO is the most classic method for building a CCH model in rats, in which both sides of bilateral common carotid arteries of rats are permanently ligated at the same time, causing ischemic-hypoxic damage in vulnerable areas of brain tissue (Jiwa et al. 2010). However, there is a high postoperative mortality rate caused by the rapid decrease of blood flow, which does not reflect the true pathological characteristics of the CCH state in clinical studies (Mansour et al. 2018). Besides, the occlusion of ophthalmic arteries after BCCAO impairs visual function in rats, which may influence rats’ behavior in behavioral experiments (Wang et al. 2016; Washida et al. 2019). As a result, many modified BCCAO procedures have been developed, such as the two-step method, unilateral ligation combined with contralateral stenosis with the help of a blunt 29-gauge needle (Sarti et al. 2002; Mansour et al. 2018). In our study, a two-step BCCAO procedure was used, in which the unilateral common carotid artery was ligated first and the contralateral common carotid artery was ligated one week later. This two-step BCCAO procedure was first used by Sarti et al. in Wistar rats, which reduced one-time injury and owned low mortality (only one died in eighteen rats) (Sarti et al. 2002).

By using PET technology, we verified that the model was successful and found that CBF changes in different brain regions were not identical in mBCCAO rats. There were obvious changes in CBF in the hippocampus, thalamus, and motor and sensory cortex regions, which was consistent with the conclusions of other similar studies (Iwasaki et al. 1989; Wang et al. 2020). Differences in angiogenesis and vascular remodeling capacity in different regions may explain this phenomenon (Choy et al. 2006), as Jun et al. have proved that the expression levels of vascular endothelial growth factor (VEGF) are different in the cortex and hippocampus under CCH state (Jun et al. 2020).

BBB, consisting of pericytes, vascular endothelial cells, basement membrane, and astrocyte terminal foot, is an integral part of the neurovascular unit (Liebner et al. 2018; Wong et al. 2019). As a result of CCH, reduced CBF leads to excitotoxicity, inflammation, oxidative stress, and overexpression of matrix metalloproteinase, all of which disrupt the integrity of the BBB (Rajeev et al. 2022). The BBB injury induces a series of downstream events, such as astrocyte proliferation, microglia activation, oligodendrocyte apoptosis, and white matter lesions, which lead to secondary brain damage (Washida et al. 2019). BBB leakage has also been identified as an important pathological change in VCI patients receiving magnetic resonance imaging (Taheri et al. 2011; Munoz Maniega et al. 2017; Wardlaw et al. 2017; Li et al. 2021). Therefore, early intervention in BBB injury is important for subsequent white matter and neuronal protection.

DL-3-n-butylphthalide (NBP) has been validated for cognitive function improvement in many animal models of CCH (Xiong et al. 2017; Qi et al. 2018; Han et al. 2019; Li et al. 2019, 2020; Niu et al. 2019; Ye et al. 2019), but most of the existing studies focus on its direct protective effects on neurons (Xiong et al. 2017; Qi et al. 2018; Li et al. 2019; Niu et al. 2019), as well as its mechanisms in promoting angiogenesis (Xiong et al. 2017; Niu et al. 2019), inhibiting neuroinflammation (Han et al. 2019; Li et al. 2020), and reducing reactive astrocyte proliferation (Xiong et al. 2017; Han et al. 2019), while the number of studies about the effect of NBP on BBB is very limited (Ye et al. 2019).

Tight junction proteins (TJs) are macromolecular complexes composed of transmembrane proteins, cytoplasmic adhesion proteins and cytoskeletal proteins, which play an essential part in maintaining the integrity of BBB. TJ proteins mainly include two broad categories: cytoplasmic zonula occludens (ZO), and three transmembrane proteins—claudins, occludins, and junctional adhesion molecules (JAMs) (Hawkins and Davis 2005). Claudin-5 is the most abundant member of the claudin family in BBB and governs selective permeation of the TJ by regulating the paracellular permeation of small molecules (Haseloff et al. 2015). ZO-1 belongs to the membrane-associated guanylate kinase-like proteins family, interacts with occludins and claudins, anchoring TJ to the cytoskeletal scaffold and actin of endothelial cells (Huber et al. 2001), and is sensitive to the BBB damage in pathological conditions, which can be used as a good indicator of the structure and function of BBB. In other CCH models, a decrease of ZO-1 and Claudin-5 has also been observed (Pan et al. 2015; Toyama et al. 2018). In clinical practice, ZO-1 and Claudin-5 are also often used as markers for BBB alteration, the levels of which are positively associated with disease prognosis (Viggars et al. 2011; Zhu et al. 2017a).

In this study, we found that NBP improved EB leakage and reduced the loss of ZO-1 and Claudin-5 in the mBCCAO rats with a dose-dependent effect, which has also been reported by Ye et al. in the traditional BCCAO model (Ye et al. 2019). The high-dose NBP was effective, while the low-dose group did not reflect significant improvements in cognitive function and the blood–brain barrier. The clinical dose for oral administration of butylphthalide is 0.2 g, *tid*, which is higher than 40 mg/kg·d and lower than 80 mg/kg·d based on the conversion relationship between oral administration in rats and humans, which may explain the reason for the ineffectiveness of low-dose NBP. It is worth noting that NBP has side effects, long-term administration of which can result in mild elevation of transaminases and mild gastrointestinal symptoms. However, previous randomized double-blind controlled trials have confirmed that NBP-related adverse events are uncommon (Jia et al. 2016).

During embryonic development, pericytes are recruited by endothelial cells to facilitate neovascularization and are involved in the regulation of angiogenesis, vascular maturation and vascular permeability (Daneman et al. 2010; Sweeney et al. 2016). The pericyte coverage ratio determines the relative permeability of vasculature, and it has been shown that there is a significant decrease in pericyte coverage in the corpus callosum in the BCCAO rat model (Sun et al. 2021). However, in our study, we found no difference in cortex and corpus callosum (data unshown) pericyte coverage between the sham and model groups. We guess, the underlying reason could be that the ischemic impairment in mBCCAO was more slight than in BCCAO, and the manual counting error could also play a part.

There are some limitations of this study. First, our experiment only explored the protective effect of NBP on BBB under the CCH state at several individual time points. It is meaningful to extend the experimental time and set more time points to observe the efficacy of NBP in the chronic process. Second, further investigations are needed to find the possible molecular mechanisms by which NBP affects TJ proteins expression after CCH. Existing studies indicated that NBP may ameliorate the loss of TJ proteins through the downregulation of autophagy levels in human brain microvascular endothelial cells in the oxygen–glucose deprivation model (Wu et al. 2020). The HIF-1α/MMP signaling pathway, which is related to the expression of tight junction proteins, has been reported to be suppressed by NBP in the BCAS model (Che et al. 2023). Third, inflammatory markers might play a part in NBP’s positive effect on the cerebral endothelium, which needs to be further explored. As reported recently in a clinical study, NBP combination with Edaravone treatment significantly decreased serum Tumor necrosis factor-α (TNF- α), C-reactive protein (CRP), and Interleukin-6 (IL-6) levels of ischemic stroke patients (Li et al. 2023). NBP has also been reported to reduce the inflammatory response induced by lipopolysaccharide by downregulating the mRNA level of pro-inflammatory cytokines Interleukin-1β (IL-1β) and IL-6 in rats’ hippocampus (Yang et al. 2018).

## Conclusion

In this article, we successfully established a CCH model using the mBCCAO procedure and evaluate CBF change in different brain regions after mBCCAO. More significant differences were detected in the hippocampus, thalamus, and motor and sensory cortex regions. Compared with the sham group, CCH rats showed cognitive dysfunction with reduced spatial learning and memory abilities. High-dose NBP (80 mg/kg) had a positive effect on improving vascular cognitive impairment of CCH rats. Moreover, the BBB disruption was attenuated by high-dose NBP treatment. In the mBCCAO model, the cortex pericyte coverage ratio didn't change, and NBP has no impact on the cortex pericyte coverage ratio. This may be due to that NBP protected the integrity of BBB by upregulating TJ protein expression, rather than regulating the pericyte coverage ratio.

## Data Availability

The original contributions presented in the study are included in the article, further inquiries can be directed to the corresponding authors.

## Abbreviations

AD: Alzheimer's disease
CBF: Cerebral blood flow
BBB: Blood-brain barrier
BCAS: Bilateral common carotid artery stenosis
CCH: Chronic cerebral hypoperfusion
CRP: C-reactive protein
IL-1β: Interleukin-1β
IL-6: Interleukin-6
LCCAO: Left common carotid artery occlusion
mBCCAO: Modified bilateral common carotid artery occlusion
MPP^+^: 1-Methyl-4-phenylpyridinium
NBP: DL-3-n-butylphthalide
PET: Positron Emission Computed Tomography
RCCAO: Right common carotid artery occlusion
SPF: Specific pathogen-free
TNF-α: Tumor Necrosis Factor-α
VaD: Vascular dementia
VCI: Vascular cognitive impairment
