# Correction to: Effects of Dl-3‑n‑butylphthalide on cognitive functions and blood–brain barrier in chronic cerebral hypoperfusion rats

**DOI:** 10.1007/s00210-023-02580-9

**Published:** 2023-06-22

**Authors:** Yang Ma, Shiling Chen, Yuanwei Li, Jiahui Wang, Jingfei Yang, Jie Jing, Xia Liu, Yunjie Li, Jingyi Wang, Ping Zhang, Zhouping Tang

**Affiliations:** 1 Department of Neurology, Tongji Hospital, Tongji Medical College, Huazhong University of Science and Technology, Wuhan, China; 2 Department of Neurology, Changzhou No.2 People’s Hospital, the Affliated Hospital of Nanjing Medical University, Changzhou, China

**Correction to** **:** **Naunyn–Schmiedeberg’s Archives of Pharmacology**

**https://doi.org/10.1007/s00210-023–02530-5**

In the published version, the second affiliation of the author Yang Ma is incorrect. The second affiliation should be corrected as “Department of Neurology, Changzhou No.2 People’s Hospital, the Affiliated Hospital of Nanjing Medical University, Changzhou, China”.

